# Myocardial perfusion is impaired in asymptomatic renal and liver transplant recipients: a cardiovascular magnetic resonance study

**DOI:** 10.1186/s12968-015-0166-5

**Published:** 2015-07-10

**Authors:** Susie Parnham, Jonathan M. Gleadle, Darryl Leong, Suchi Grover, Craig Bradbrook, Richard J. Woodman, Carmine G. De Pasquale, Joseph B. Selvanayagam

**Affiliations:** Department of Cardiovascular Medicine, Flinders Medical Centre, Bedford Park, Adelaide, SA 5042 Australia; Department of Renal Medicine, Bedford Park, Adelaide, SA Australia; School of Medicine, Flinders University, Bedford Park, Adelaide, SA Australia; Flinders Centre for Epidemiology and Biostatistics, School of Medicine, Flinders University, Bedford Park, Adelaide, SA Australia; Population Health Research Institute, Hamilton, ON Canada

**Keywords:** Magnetic resonance, Renal transplant, Coronary artery disease, Myocardial perfusion, Liver transplant

## Abstract

**Background:**

Myocardial ischemia is a major cause of death in chronic kidney disease (CKD) patients, which can be caused by either epicardial or microvascular coronary artery disease (CAD). Although renal transplantation improves survival, cardiovascular disease remains a major cause of mortality in post renal transplant recipients, including those with no significant epicardial CAD pre-transplant. We aim to utilize stress cardiovascular magnetic resonance (CMR) and MR coronary angiography (MRCA) to assess silent myocardial ischemia and epicardial CAD in renal transplant recipients.

**Methods:**

Forty-five subjects: twenty renal transplant (RT) with no known CAD, fifteen liver transplant (LT) controls without prior CKD and no known CAD, and ten hypertensive (HT) controls underwent stress perfusion CMR and MRCA.

**Results:**

A total of 1308 myocardial segments (576 of RT, 468 of LT, and 264 of HT) were compared using mixed linear modeling. Left ventricular mass index, septal diameter and presence of diabetes mellitus were similar between the groups. The mean transmural MPRI was significantly lower in the RT and LT groups compared to HT controls (1.19 ± 0.50 in RT versus 1.23 ± 0.36 in LT versus 2.04 ± 0.32 in HT controls, *p* < 0.0001), in the subepicardium (1.33 ± 0.57 in RT versus 1.30 ± 0.33 in LT versus 2.01 ± 0.30 in HT controls, *p* < 0.001), and in the subendocardium (1.19 ± 0.54 in RT versus 1.11 ± 0.31 in LT versus 1.85 ± 0.34 in HT controls, *p* < 0.0001). Seven (35 %) RT and five (33 %) LT had significant epicardial CAD compared to none in HT controls, *p* = 0.12. One RT and one LT had LGE suggesting sub-endocardial infarction.

**Conclusions:**

RT recipients have impaired myocardial perfusion independent of LVH or diabetes mellitus. The impaired myocardial perfusion in RT is similar to LT without prior renal disease, thus unlikely related to previous CKD. It is not fully explained by the presence of significant epicardial CAD, and therefore most likely represents microvascular CAD.

## Background

Cardiovascular disease is the leading cause of mortality and morbidity in the chronic kidney disease (CKD) population, accounting for 50 % of all deaths [1]. CKD patients have a 10 to 20 fold increased risk of cardiac death than the normal population, although the mechanism is uncertain [1]. Furthermore, despite the risk of cardiovascular mortality being significantly reduced by renal transplantation [2], cardiovascular disease remains a major cause of mortality in post-renal transplant recipients with an annual event rate of 3.5 to 5 % [3]. Renal transplant recipients carry a multitude of risk factors including traditional atherosclerotic (dyslipidemia, hypertension, diabetes), myocardial (hypertension and fluid overload pre transplant), microvascular (diabetes, renal failure), and immunosuppression.

Current diagnostic investigations of myocardial ischemia in renal population lack sensitivity and specificity or may have adverse effects [4]. Multi-parametric Cardiovascular Magnetic Resonance (CMR) enables concurrent assessment of myocardial function, perfusion and irreversible injury with high spatial resolution [5]. In particular, stress perfusion CMR has high sensitivity and negative predictive value for detecting myocardial ischemia with a sensitivity of 89 % and a specificity of 80 % [6]. In addition, two large prospective controlled trials,-CE-MARC and MR-IMPACT II- demonstrated higher diagnostic accuracy of stress perfusion CMR compared to SPECT [7, 8]. Huber et al. found that semi-quantitative evaluation provides identical diagnostic performance for coronary artery disease (CAD) to quantitative evaluation if both stress and rest examinations were used [9]. Recently, magnetic resonance coronary angiography (MRCA) has emerged as an imaging alternative for coronary artery anatomy, especially for the proximal and mid coronary segments [10]. Finally, CMR allows accurate quantification of ventricular function and mass as well as tissue characterization, thereby uniquely positioning it as a powerful modality to explore the high cardiovascular event rate in renal transplant patients.

We sought to investigate the mechanism of cardiovascular morbidity and mortality in otherwise well post renal transplant recipients using multi-parametric CMR. Our primary aim was to assess the presence and degree of myocardial ischemia utilizing stress perfusion CMR and the presence of significant epicardial disease using non-contrast whole-heart MRCA. Majority (50–90 %) of renal transplant recipients have hypertension [11] and the high (75 %) prevalence of left ventricular hypertrophy (LVH) in CKD population [12] likely persists post transplantation [13]. In contrast to the advanced CKD pre-renal transplant population, the prevalence of CAD in the end-stage liver disease patients is similar or only slightly greater than the normal population, ranging from 2.5 % to 27 %, however, cardiovascular disease is a major cause of mortality post liver transplantation [14]. We used 2 control groups- an aged matched population of hypertensive controls (to control for LVH commonly seen in the renal transplant recipients) and a post-liver transplant group (to allow differentiation of transplant milieu effects from prior renal failure effects).

## Methods

### Study population

Renal transplant (RT) recipients who were well and with stable renal function between three months and five years post transplantation were invited to participate to have CMR imaging at Flinders Medical Centre, a tertiary teaching hospital in South Australia, in 2012–2014. RT subjects had the following inclusion criteria: no established CAD (no history of myocardial infarction, angina, coronary artery stent or bypass surgery or angiographically documented significant CAD > 70 %, and no significant inducible myocardial ischemia pre-transplant), and no previous systolic heart failure. Liver transplant (LT) recipients with the same inclusion criteria were recruited. Ten people with a clinical diagnosis of hypertension (HT) and who were asymptomatic with no known CAD were prospectively recruited from the hospital’s Hypertension Clinic.

Exclusion criteria for each group were severe claustrophobia, metallic implants, contraindications to adenosine (second or third degree atrioventricular block, obstructive pulmonary disease, dipyridamole use), and contraindications to gadolinium chelate (anaphylaxis, estimated glomerular filtration rate (eGFR) < 45 ml/min/1.73 m^2^).

We identified a total of 171 RT patients from the hospital’s renal database within the calendar year 2012. Exclusions were: >60 months post renal transplantation (102), ischaemic heart disease or known coronary artery disease (11), CMR contraindication (20), <3 months post renal transplantation (2), declined participation (13), language barrier (2) and pregnancy (1). Following exclusion, a total of 20 RT patients were enrolled into the study.

All participants gave written informed consent, and the study was approved by Southern Adelaide Clinical Human Research Ethics Committee (SAC HREC).

### CMR protocol

All participants were instructed to refrain from caffeine 24 h prior to the scan. Subjects on beta blockers continued with their medications.

Cine imaging was acquired using standard method [15]. Stress imaging with adenosine infusion 140 μg/kg/min for 3–4 min was performed of the basal, mid, and apical myocardial segments, using an ECG-gated T1-weighted fast gradient echo sequence (echo time, 1.04 ms; repetition time, 2 ms; voxel size, 29×2.3×8 mm, flip angle 17°), and a peripheral bolus injection of a gadolinium-based agent (0.1 mmol/kg; gadolinium-based contrast agent, Gadovist, Bayer, Australia), followed by a 15 ml bolus of normal saline (rate 5 ml/s), as previously described [16, 17]. All slices were imaged during each heart beat, for a total of 50 heart beats. Blood pressure and heart rate were recorded by an automated recording machine at baseline and at 1 min intervals during adenosine infusion. After discontinuing adenosine for 15 min, the same sequence was repeated without intravenous adenosine to obtain resting perfusion images. For late enhancement imaging, an additional bolus of Gadovist (0.05 mmol/kg) was injected, and after 6 min, images were acquired in the 3 long axes and in the short axis plane to obtain coverage of the entire left ventricle using a gated T1-weighted segmented inversion recovery turbo fast low-angle shot sequence (echo time, 4.8 ms; voxel size, 1.4×2.4×8 mm; flip angle, 20°). The inversion time was adjusted to achieve optimal nulling of non-infarcted myocardium, as previously described [18].

MRCA images were obtained as a separate scan by using an 18-channel flex coil 1.5 T clinical MR scanner (Siemens Sonata, Erlangen, Germany). A four-lead ECG was obtained for cardiac gating. Glyceryl trinitrate 400 micrograms/metered dose was administered prior to MRCA. The navigator-gated, free-breathing, non-contrast whole-heart CMRA was acquired using a 3D segmented Steady-State Free Precession sequence protocol as previously described [19, 20].

### CMR image analysis

CMR analysis was performed with CMR^42^ Version 4.1, Circle Cardiovascular Imaging Inc. Left ventricular mass, left and right ventricular volumes and functions were calculated using 3D short axis stack by tracing of the endocardial and epicardial contours in end-diastole and end-systole, as previously described [15]. Each parameter was indexed to body surface area (BSA). The septal and lateral wall diameters were measured in end-diastole at mid-ventricular level from short-axis view.

**Fig. 1:**
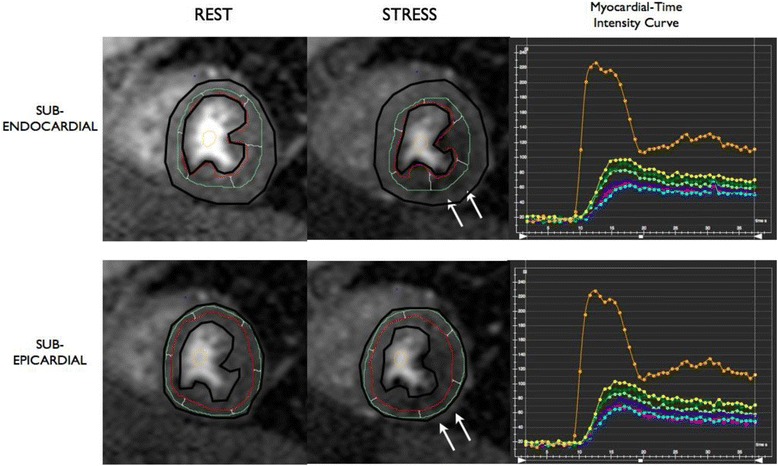
Example of Subendocardial and Subepicardial Segmentation. Rest and stress perfusion images of a transplant recipient showing inferior and anterolateral perfusion defect (white arrows). Basal, mid, and apical short axis slices were acquired (a mid short axis slice was shown as representative). Transmural, subepicardial (bottom) and subendocardial (top) contours were traced. The left ventricular myocardium was segmented into six segments (anterior, anterolateral, inferolateral, inferior, inferoseptal, anteroseptal) with right ventricular insertion point as a reference. Segmental myocardial-time intensity curves at stress (shown on the right) and at rest were measured by the CMR^42^ software

For perfusion analysis, semi-quantitative analysis using CMR^42^ software was used. Transmural, subepicardial and subendocardial contours were traced and manually corrected for breathing displacement (Fig. 1). Each basal, mid and apical myocardial slice were divided into 6 segments with the right ventricular insertion as the reference point [21]. Since basal myocardial blood flow is closely related to the rate-pressure product (RPP), and index of left ventricular oxygen consumption, values for rest flow in each patient were also corrected for rate-pressure product [22]: Corrected Rest perfusion = (Rest perfusion/RPP) × 10^4^. Myocardial Perfusion Reserve Index (MPRI) was calculated as the ratio of perfusion during adenosine-induced hyperemia to perfusion at rest corrected for RPP [23].

For late enhancement analysis, areas of subendocardial hyperenhancement were assessed visually as present or absent. The fibrosis was quantified using CMR^42^ software as a percentage of myocardial mass [24, 25].

MRCA images were transferred to a 4D viewer image reconstruction CMR^42^ software. Three-dimensional volume-rendered images were generated by the software. The left main, left anterior descending artery (LAD), left circumflex (LCx) and right coronary artery (RCA) were manually traced and followed for their course in axial, coronal, sagittal, cranial left anterior oblique, lateral, caudal right anterior oblique and caudal left anterior oblique views. The coronary arteries were analysed and segmented according to the American College of Cardiology/American Heart Association classification [26].

One CMR experienced cardiologist (JBS) blinded to the clinical information and stress perfusion results, evaluated the left main, LAD, LCx and RCA arteries using sliding thin-slab maximum intensity projection. Significant coronary artery stenoses were defined as luminal narrowing greater than 50 % [20]. Minor coronary artery disease was defined as luminal narrowing of less than 50 %. The coronary artery was classified normal if it was smooth without any plaque occupying lumen.

## Statistical analysis

Statistical analysis was performed with STATA version 13.0. Parametric data is expressed as mean ± SD and non-parametric values as median (inter-quartile range). Independent t-tests and ANOVA was used to compare the clinical characteristics of the study groups. Fisher’s exact test was used for comparison of categorical variables. MPRI evaluation of coronary artery level data was analysed using linear mixed modeling (LMM) with a random intercept used for each subject to account for the within-subject correlation present from measuring at 3 different artery sites. Both unadjusted and adjusted LMM was performed with adjustment for medication use (where significant in univariate analysis) and left ventricular mass a priori. Statistical tests were 2-tailed and a *p* value <0.05 was considered statistically significant.

## Results

### Subject characteristics

Forty-five subjects participated in the study: twenty RT, fifteen LT controls, and ten HT controls participated in the study. Clinical characteristics are presented in Table 1. Using Bonferroni correction for multiple group comparison, the eGFR was lower in the renal transplant group compared to hypertensive control (*p* = 0.012), but similar compared to liver transplant group (*p* = 0.67). The eGFR was similar between the liver transplant and hypertensive control groups (*p* = 0.21). The presence of hypertension was similar between the renal transplant and hypertensive groups. The liver transplant group had less degree of hypertension compared to the hypertensive group (*p* = 0.027). The presence of hypertension in the renal transplant and liver transplant groups was not statistically different (*p* = 0.06). The use of statin between the renal transplant and hypertensive groups was similar (*p* = 0.95).

**Table 1:** Clinical characteristics

	Renal transplant subjects (*n* = 20)	Liver transplant subjects (*n* = 15)	Hypertensive controls (*n* = 10)	*p*-value*
Age, years (mean ± SD)	55 ± 11	61 ± 6	55 ± 11	0.17
Male sex, *n* (%)	11 (55)	12 (80)	5 (50)	0.18
BMI^a^, kg/m^2^ (mean ± SD)	29 ± 5	30 ± 4	33 ± 3	0.26
eGFR^b^, mL/min/1.73 m^2^ (mean ± SD)	78 ± 19	89 ± 29	108 ± 30	0.009
Systolic Blood Pressure (mmHg)	131 ± 19	130 ± 14	140 ± 11	0.26
Diastolic Blood Pressure (mmHg)	77 ± 14	80 ± 9	80 ± 11	0.74
Heart Rate (beats per minute)	71 ± 11	64 ± 9	77 ± 15	0.02
Cardiovascular Risk Factors, *n* (%)				
Hypertension	18 (90)	9 (60)	10 (100)	0.03
Diabetes Mellitus	3 (15)	4 (27)	2 (20)	0.62
Total cholesterol (mmol/L)	4.8 ± 1.2	4.5 ± 1.1	5.7 ± 1.0	0.06
Low-density lipoprotein (mmol/L)	2.1 ± 1.1	2.6 ± 1.0	3.6 ± 0.9	0.005
Triglyceride (mmol/L)	2.3 ± 1.4	1.5 ± 0.6	1.5 ± 0.7	0.08
Smoking History	7 (35)	6 (40)	3 (30)	1.00
Cardiac Medications, n (%)				
Aspirin	1 (5)	0 (0)	2 (20)	0.14
Beta blocker	9 (45)	3 (20)	2 (20)	0.29
ACE^c^ inhibitor	5 (25)	1 (7)	2 (20)	0.41
Angiotensin Receptor Blocker	3 (15)	1 (7)	5 (50)	0.07
Calcium channel blocker	5 (25)	5 (33)	5 (50)	0.54
Statin	7 (35)	0 (0)	2 (20)	0.03

Data are presented as n (%) or mean ± SD

*Assessed using ANOVA or Fisher’s exact as appropriate

^a^BMI indicates body mass index

^b^eGFR, estimated Glomerular Filtration Rate

^c^ACE, angiotensin-converting enzyme

The etiology of renal diseases in the RT group were: polycystic kidney disease (*n* = 7), glomerulonephritis (*n* = 9), diabetic nephropathy (*n* = 1), medication related (*n* = 1), reflux nephropathy (*n* = 1), Alport’s syndrome (*n* = 1), and unknown (*n* = 1). The etiology of liver diseases in the LT group were: alcoholic liver disease (*n* = 7), hepatitis C (*n* = 5, 1 of which was combined hepatitis C and B), primary sclerosing cholangitis (*n* = 1), non-alcoholic steatohepatitis (*n* = 1), and unknown (*n* = 1). The mean post-transplant duration between the RT and LT groups were similar (33 ± 17 versus 36 ± 18 months, *p* = 0.44). The immunosuppressant medications received by the RT and LT groups are outlined in Table 2. Their exposures were similar apart from LT patients only receiving prednisolone in the first three months post transplantation and the use of mycophenolate, which was more prevalent in RT patients.

**Table 2:** Prescribed immunosuppressant medications in the renal and liver transplant groups

	Renal transplant subjects (*n* = 20)	Liver transplant subjects (*n* = 15)	*p*-value*
Immunosuppressant, *n* (%)			
Azathioprine	2 (10)	4 (27)	0.37
Mycophenolate	16 (80)	4 (27)	0.002
Prednisolone	18 (90)	0 (0)	<0.0001
Cyclosporine	1 (45)	1 (7)	0.68
Tacrolimus	16 (80)	14 (93)	0.37
Everolimus	1 (5)	0 (0)	0.57
Sirolimus	1 (5)	0 (0)	0.57

*Asessed using Fisher’s exact

### Assessment of left ventricular mass, volumes and function

The CMR results are summarized in Table 3. Left ventricular mass index, septal and lateral wall diameter were similar between the groups.

**Table 3:** Left ventricular mass, septal and lateral wall thickness, ventricular volumes and ejection fraction

	Renal transplant subjects (*n* = 20)	Liver transplant subjects (*n* = 15)	Hypertensive controls (*n* = 10)	*p*-value*
LV^a^ Mass index, g/m^2^	64 ± 13	65 ± 11	60 ± 10	0.60
LV Septal Wall thickness, cm	1.2 ± 0.3	1.2 ± 0.2	1.1 ± 0.3	0.22
LV Lateral Wall thickness, cm	0.9 ± 0.3	0.9 ± 0.2	0.9 ± 0.2	0.81
LV End Diastolic Volume index, ml/m^2^	68 ± 12	62 ± 13	70 ± 13	0.43
LV End Systolic Volume index, ml/m^2^	18 ± 8	19 ± 8	22 ± 9	0.75
LV Stroke Volume index, ml/m^2^	50 ± 12	45 ± 9	46 ± 10	0.26
LV Ejection Fraction, %	74 ± 9	71 ± 8	69 ± 8	0.45
RV^b^ End Diastolic Volume index, ml/m^2^	70 ± 11	67 ± 14	71 ± 18	0.73
RV End Systolic Volume index, ml/m^2^	27 ± 8	24 ± 6	26 ± 9	0.47
RV Stroke Volume index, ml/m^2^	43 ± 8	41 ± 11	46 ± 12	0.77
RV Ejection Fraction, %	62 ± 10	63 ± 7	63 ± 6	0.92

All data are presented as mean ± SD

*Assessed using ANOVA

^a^LV indicates Left Ventricle

^b^RV, Right Ventricle

### Assessment of Myocardial Perfusion Reserve Index (MPRI)

A total of 1308 out of 1452 (90 %) myocardial segments were analyzable. A total of 576 of RT, 468 of LT, and 264 of HT) were analyzed and compared using mixed linear modeling.

**Fig. 2:**
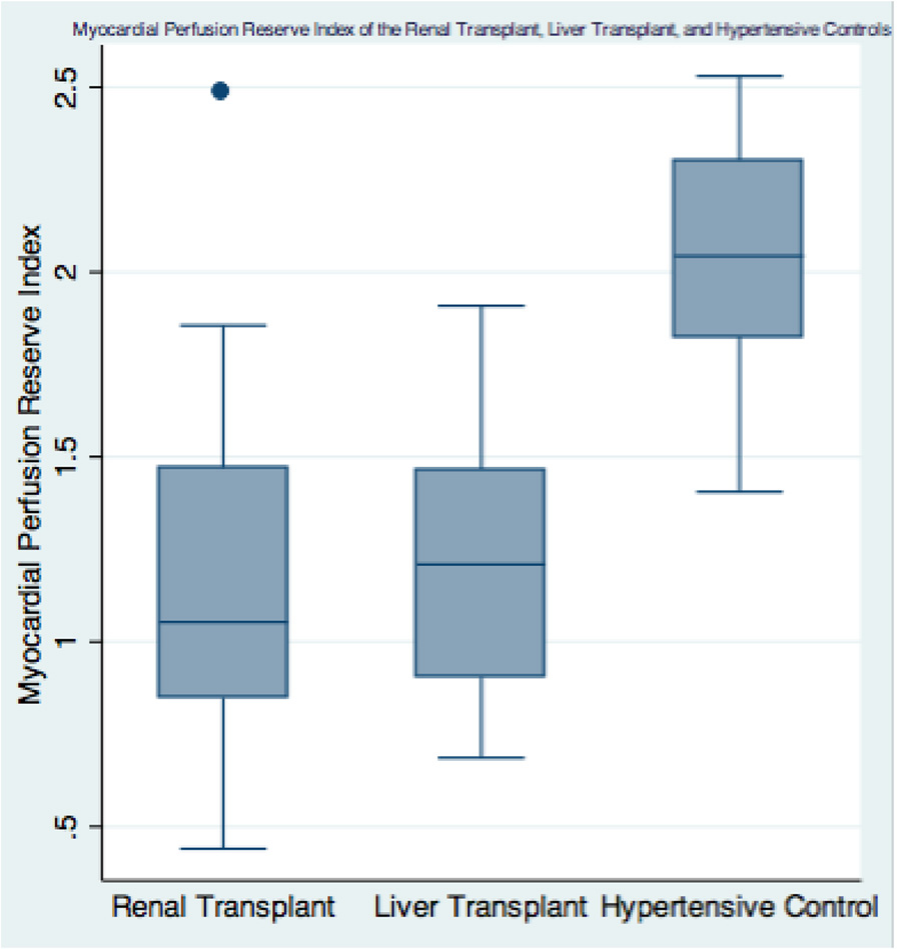
Mean Myocardial Perfusion Reserve Index (MPRI) of the Renal Transplant, Liver Transplant, and Hypertensive Controls (1.19 ± 0.50 in RT versus 1.23 ± 0.36 in LT versus 2.04 ± 0.32 in HT controls, *p* < 0.0001)

**Fig. 3:**
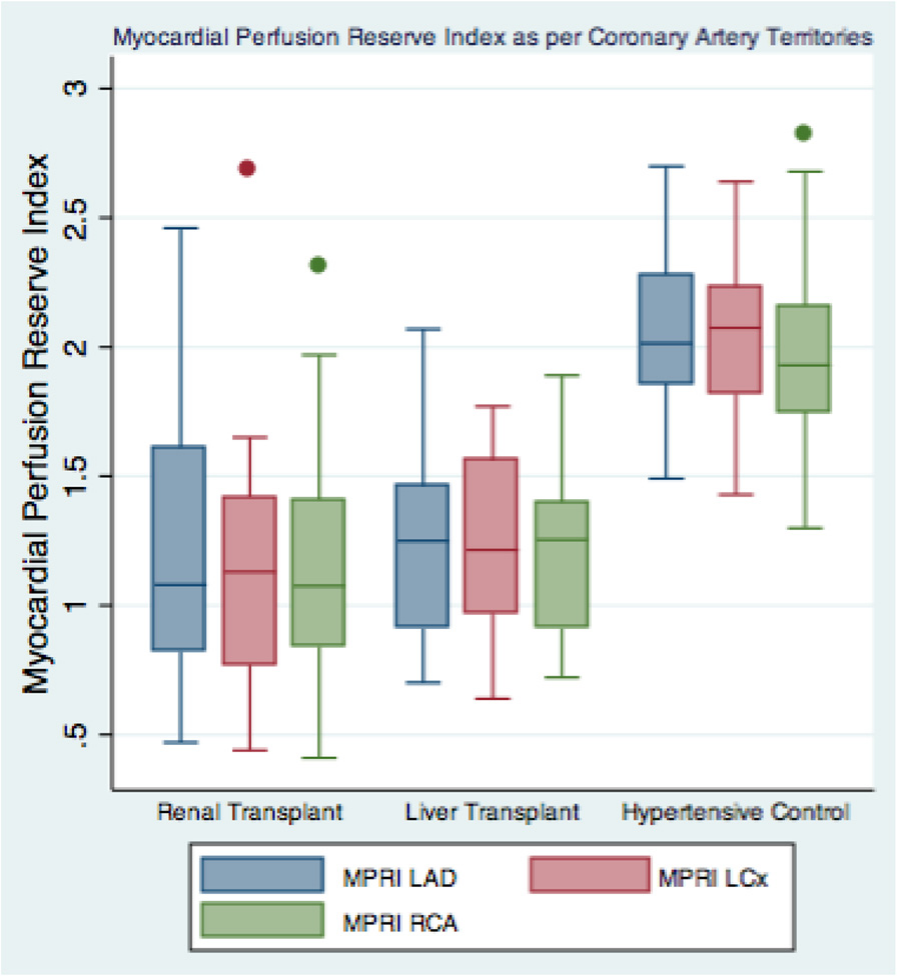
Myocardial Perfusion Reserve Index (MPRI) of the Renal Transplant, Liver Transplant, and Hypertensive Controls within each of the three coronary artery territories LAD indicates Left Anterior Descending; LCx, Left Circumflex; RCA, Right Coronary Artery

**Fig. 4:**
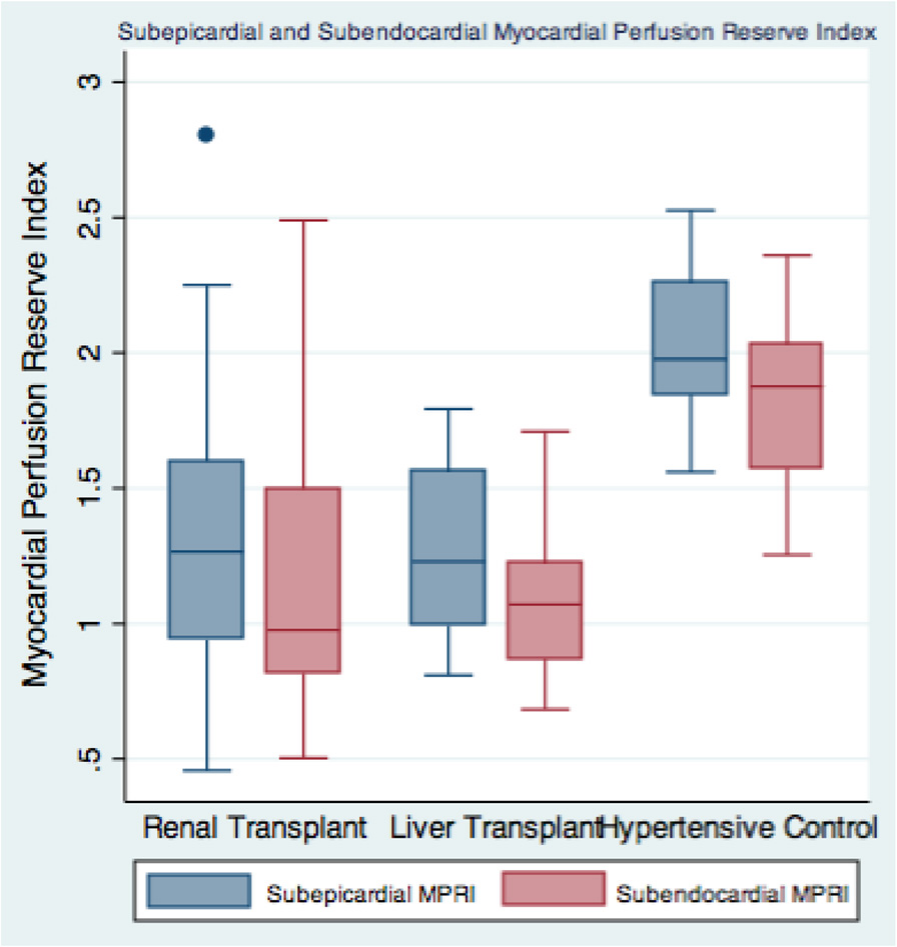
Subepicardial and Subendocardial Myocardial Perfusion Reserve Index (MPRI) of the Renal Transplant, Liver Transplant, and Hypertensive Controls (Subepicardial MPRI 1.33 ± 0.57 in RT versus 1.30 ± 0.33 in LT versus 2.01 ± 0.30 in HT controls, *p* < 0.001; Subendocardial MPRI 1.19 ± 0.54 in RT versus 1.11 ± 0.31 in LT versus 1.85 ± 0.34 in HT controls, *p* < 0.0001)

Figures 2, 3 and 4 show the distribution of the MPRI across the three groups and within each of the three coronary artery sites. Across all 3 artery sites combined, the mean transmural MPRI was significantly reduced in RT subjects compared to HT controls but was similar between RT and LT subjects (1.19 ± 0.50 in RT versus 1.23 ± 0.36 in LT versus 2.04 ± 0.32 in HT controls, *p* < 0.0001). The subepicardial MPRI was 1.33 ± 0.57 in RT versus 1.30 ± 0.33 in LT versus 2.01 ± 0.30 in HT controls, *p* < 0.001. The subendocardial MPRI was 1.19 ± 0.54 in RT versus 1.11 ± 0.31 in LT versus 1.85 ± 0.34 in HT controls, *p* < 0.0001. In the renal transplant and liver transplant subgroups, the transmural MPRI was 0.97 ± 0.54 in those with significant CAD >50 % versus 1.05 ± 0.00 in those without, *p* = 0.02. Fifteen out of 20 (75 %) RT versus eleven out of 15 (73 %) LT versus one out of 10 (10 %) HT had transmural MPRI < 1.5 in at least one coronary artery territory, *p* < 0.0001.

Results remained similar in mixed model regression analysis after adjustment for statin use, aspirin use and left ventricular mass index with MPRI lower in RT subjects compared to HT controls (β = 0.85, *p* < 0.0001) but similar to LT subjects (β = 0.04, *p* = 0.79).

Significantly, in the RT group, the mean MPRI was associated with eGFR (β = 0.014, 95 % CI = 0.0023 to 0.026, p = 0.0019).

### Assessment of myocardial fibrosis

One RT subject had late gadolinium enhancement indicating sub-endocardial infarction in the inferoseptal wall (1.7 g of infarct mass (0.8 % of LV mass)), while one LT subject had late gadolinium enhancement indicating sub-endocardial infarction in the lateral wall (2.9 g of infarct mass (2.1 % of LV mass)).

### Assessment of epicardial coronary artery disease

**Fig. 5:**
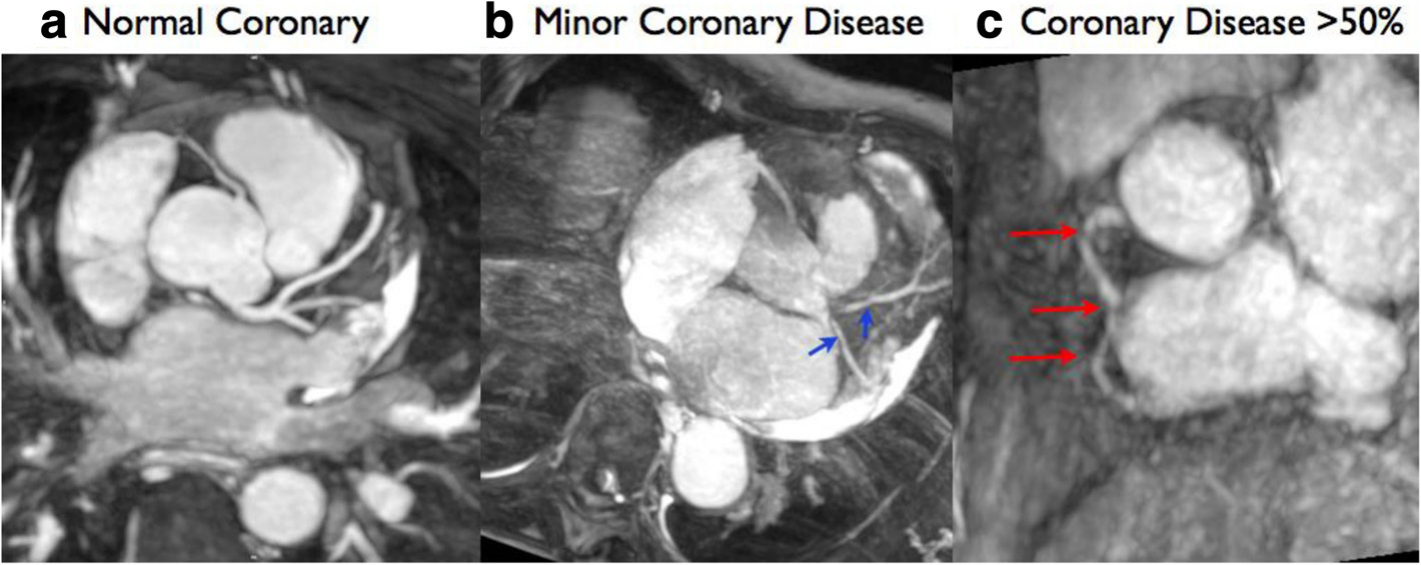
Reformatted whole-heart magnetic resonance angiography (MRCA) with navigator-gated 3D Steady-State Free Precession sequence in (**a**) transplant recipient with normal coronary arteries, (**b**) transplant recipient with minor coronary artery disease <50 % with irregularities (arrows), and (**c**) transplant recipient with coronary artery disease >50 % stenosis in RCA (arrows)

Seven out of 20 (35 %) RT, five out of 15 (33 %) LT, and 0 out of 10 (0 %) HT controls had coronary artery stenosis >50 % in at least one coronary artery territory, *p* = 0.12. There was no significant relationship between epicardial CAD and transmural MPRI as per coronary artery territories (β = −0.14, 95 % CI −0.30 to 0.23, *p* = 0.09). Figure 5 shows representative MRCA images of the transplant recipients.

## Discussion

Stress perfusion CMR and MRCA provide valuable insight into the cardiac phenotype. To our knowledge, this is the first study to investigate myocardial perfusion in asymptomatic post renal transplantation patients using stress perfusion CMR and MRCA. We have demonstrated that myocardial perfusion is significantly reduced in asymptomatic post renal transplant patients independently of the degree of left ventricular hypertrophy and diabetes mellitus. Furthermore, MRCA abnormalities do not seem to explain the perfusion abnormalities. Our findings suggest that myocardial perfusion abnormalities in renal transplant patients are predominantly due to coronary microvascular dysfunction possibly secondary to post-transplant milieu rather than significant epicardial disease. Our study findings may assist in understanding the contributors to cardiac mortality and morbidity in the post- renal transplant population, thus, may lead to better management of coronary microvascular disease in this population.

CMR stress perfusion has been well validated in the assessment of epicardial coronary disease and/or coronary microvascular dysfunction. A recent meta-analysis showed stress perfusion CMR to have a high sensitivity of 89 % and a specificity of 80 % for diagnosis of significant obstructive coronary artery disease [6]. The sensitivity and specificity of stress CMR performed with a semi-quantitative measure of myocardial perfusion reserve index (MPRI) with a cutoff value of 1.5 for the detection of functionally significant (by Fractional Flow Reserve) coronary heart disease were 91 % and 94 %, respectively, with positive and negative predictive values of 91 % and 94 % [27]. Impaired coronary flow reserve suggestive of microvascular dysfunction has been reported in pre-transplant end-stage renal disease patients without coronary artery disease [28]. Impaired coronary flow reserve, similarly, has been observed in post renal transplant patients, even at a young age [29–31]. Given the association between LVH and reduced MPRI [32], it was important that we controlled for the degree of LVH when assessing myocardial perfusion in the renal cohort. Our HT, RT and LT groups were well matched in respect of degree of LVH, LV mass, and diabetes mellitus. Hence, our finding of reduced MPRI in the RT group is unlikely to solely reflect LVH or the degree of diabetes, and likely reflects additional abnormalities in coronary microvascular function and/or asymptomatic (‘occult’) epicardial coronary artery disease.

In order to further identify the mechanisms of MPRI reduction we compared the RT group with a second control group of liver transplant patients. Pre-renal transplant CKD patients have high prevalence of cardiovascular disease, in contrast, pre-liver transplant chronic liver disease (CLD) patients have low prevalence of cardiovascular disease [33]. Whilst most CKD patients have hypertension, CLD patients have portal hypertension, which causes vasodilatation and decreased in arterial blood pressure. An et al. studied 1045 liver cirrhosis patients matched with 6283 controls and showed that asymptomatic cirrhotic patients had similar prevalence of obstructive CAD compared to controls with healthy livers [33]. However, cardiovascular disease is one of the leading cause of death in post liver transplant patients [34]. A retrospective study of 455 liver transplant recipients by Fussner et al. showed that cardiovascular disease developed in 10.6 %, 20.7 % and 30.3 % of liver transplant recipients within one, five and eight years respectively [35]. In our study, the LT and RT groups were well matched in terms of the time post-transplant, age, and duration and importantly exposure to immunosuppressive medications (except for corticosteroids and mycophenolate). Intriguingly, we found that myocardial perfusion reserve was reduced in asymptomatic post renal transplant patients similar to post liver transplant patients, despite the relatively low prevalence of CAD in chronic liver disease compared to CKD patients, and despite early discontinuation of steroid use in the latter. This finding tends to absolve corticosteroid exposure as responsible for the myocardial perfusion abnormality. In our renal transplant cohort 75 % had an MPRI < 1.5 in any coronary artery territory versus 73 % of the liver transplant cohort. Although our numbers are small, there is a strong biologic plausibility to this finding, given the well recognized effect of immunosuppressants in potentiating an increased prevalence of traditional cardiovascular risk factors in this population. Tacrolimus may cause vasoconstriction of the afferent and efferent glomerular arterioles, similar to cyclosporine [36]. The same mechanism may also induce coronary vasoconstriction or “spasm”.

In order to investigate the presence of asymptomatic epicardial CAD, we further assessed both the RT and LT groups with MRCA. This showed that 31 % of patients post transplant (both liver and renal) had significant coronary artery disease in at least one territory. We do not routinely perform invasive coronary angiography before renal or liver transplantation in our centre. Thirty-one out of thirty-five renal transplant recipients had negative stress imaging pre-transplantation. The remaining four transplant recipients had inconclusive stress imaging and underwent coronary angiography greater than five years previously that showed only minor coronary artery disease (less than 50 % in major epicardial vessel). There was no significant difference between the presence of significant CAD between RT and LT patients. Furthermore, there was no significant relationship between the presence of significant epicardial coronary artery disease and impaired myocardial perfusion reserve, implying that the mechanism of MPRI reduction is small vessel related (“coronary microvascular”) rather than epicardial disease. Microvascular CAD has been shown to be associated with reduced survival, although the rate of survival is better than for epicardial CAD [37].

Our study has several limitations. Firstly, the sample size for each group of patients was relatively small, consistent with being a pilot study. Secondly, the MRCA has lower diagnostic accuracy in the distal vessels compared with CT coronary angiography but is a safer option for post transplant recipients who have increased risk of malignancy even with small dose of radiation. Thirdly, a semi-quantitative method was used to analyze myocardial perfusion reserve since we do not have quantitative method in our center. Quantitative perfusion using CMR [17] would have permitted distinction between impaired MPRI from reduced stress myocardial blood flow (MBF) versus increased resting MBF in post transplant population. Non-contrast T1 mapping was also not available at the time of the study. Our study demonstrates the utility of multi-parametric CMR in renal transplant recipients, and confirmation in larger scale studies is warranted.

## Conclusion

Asymptomatic renal transplant recipients have a global reduction in myocardial perfusion, independent of the degree of LVH and the presence of diabetes mellitus. Myocardial perfusion is also impaired in liver transplant recipients, thus unlikely due to previous CKD. In our transplant cohort, the impaired myocardial perfusion is incompletely accounted for by epicardial coronary artery disease suggesting a pathophysiologic role for coronary microvascular dysfunction in this clinical setting.

## Abbreviations

CAD: Coronary artery disease
CKD: Chronic kidney disease
CMR: Cardiovascular magnetic resonance
eGFR: Estimated glomerular filtration rate
HT: Hypertensive
LVH: Left ventricular hypertrophy
LT: Liver transplant
MPRI: Myocardial Perfusion Reserve Index
RT: Renal transplant
